# Leucocyte and Platelet‐rich Fibrin: a carrier of autologous multipotent cells for regenerative medicine

**DOI:** 10.1111/jcmm.13468

**Published:** 2018-01-05

**Authors:** Rosa Di Liddo, Thomas Bertalot, Alessio Borean, Ivan Pirola, Alberto Argentoni, Sandra Schrenk, Carola Cenzi, Stefano Capelli, Maria Teresa Conconi, Pier Paolo Parnigotto

**Affiliations:** ^1^ Department of Pharmaceutical and Pharmacological Sciences University of Padova Padova Italy; ^2^ Foundation for Biology and Regenerative Medicine Tissue Engineering and Signaling (TES) ONLUS Padova Italy; ^3^ Department of Immunohematology and Transfusion Medicine San Martino Hospital Belluno Italy; ^4^ Department of Chemistry and Technology of Drugs Sapienza University of Rome Italy

**Keywords:** circulating multipotent cells, haemoderivatives, *in vivo* guided regeneration, autologous cell therapies

## Abstract

The wound healing is a complex process wherein inflammation, proliferation and regeneration evolve according to a spatio‐temporal pattern from the activation of coagulation cascade to the formation of a plug clot including fibrin matrix, blood‐borne cells and cytokines/growth factors. Creating environments conducive to tissue repair, the haemoderivatives are commonly proposed for the treatment of hard‐to‐heal wounds. Here, we explored *in vitro* the intrinsic regenerative potentialities of a leucocyte‐ and platelet‐rich fibrin product, known as CPL‐MB, defining the stemness grade of cells sprouting from the haemoderivative. Using highly concentrated serum‐based medium to simulate wound conditions, we isolated fibroblast‐like cells (CPL‐CMCs) adhering to plastic and showing stable *in vitro* propagation, heterogeneous stem cell expression pattern, endothelial adhesive properties and immunomodulatory profile. Due to their blood derivation and expression of CXCR4, CPL‐CMCs have been suggested to be immature cells circulating in peripheral blood at quiescent state until activation by both coagulation event and inflammatory stimuli such as stromal‐derived factor 1/SDF1. Expressing integrins (CD49f, CD103), vascular adhesion molecules (CD106, CD166), endoglin (CD105) and remodelling matrix enzymes (MMP2, MMP9, MMP13), they showed a transendothelial migratory potential besides multipotency. Taken together, our data suggested that a standardized, reliable and economically feasible blood product such as CPL‐MB functions as an artificial stem cell niche that, under permissive conditions, originate *ex vivo* immature cells that could be useful for autologous stem cell‐based therapies.

## Introduction

Over the last three decades, the enormous progress in cell processing technology has enhanced a general shift from heterologous to autologous stem cell‐based therapies. In the prospect of having biomaterials and bioactive surgical additives with predictable outcome in regenerative medicine, several techniques have been developed to process peripheral blood and to obtain products useful for controlling inflammation and enforcing the physiological events of haemostasis and wound healing 1, 2, 3, 4. Depending on their contents of platelets, leucocytes and fibrin architecture, they are commonly classified into four families: (*i*) pure platelet‐rich plasma (P‐PRP, in liquid or gel form); (*ii*) leucocyte‐ and platelet‐rich plasma (L‐PRP, in liquid or gel form); (*iii*) pure platelet‐rich fibrin (P‐PRF); and (*iv*) leucocyte‐ and platelet‐rich fibrin (L‐PRF) 5. Among them, L‐PRF offers overall higher amounts of released TGF‐β1, a sustained, long‐term release of growth factors (VEGF, IGF1, PDGF‐AB) and cytokines (IL‐1β), and stronger induction of cell migration *in vitro* 6. Obtained by different production methods and devices, the haemoderivatives demonstrate to be beneficial for tissues with restricted blood supply, slow cell turnover, limited extracellular matrix restoration facilitating the recruitment, proliferation and maturation of cells participating in regeneration. They are commonly used in clinics for numerous medical applications including (*i*) the healing of recalcitrant ulcers and burns; (*ii*) the stimulation of tissue regeneration in dentistry, implantology, and maxillofacial and plastic surgery; (*iii*) the treatment of knee osteoarthritis; and (*iv*) the repair of musculoskeletal tissue, tendon, and ligament lesions 1, 7, 8. To date, the intrinsic regenerative potentialities of L‐PRP have been commonly attributed to platelet‐ and leucocyte‐derived factors (coagulation factors, growth factors and cytokines) and fibrin matrix that synergistically orchestrate the recruitment of stem cells or progenitor cells following an inflammatory response driven by neutrophils, M1‐polarized macrophages and T lymphocytes (early‐phase), and M2 macrophages (late‐phase) 9. A growing body of evidence demonstrates that the contribution of L‐PRF to *in vivo* angiogenesis and vasculogenesis at injury site is mediated by intrinsically carried haematopoietic stem cells (HSCs) (CD34^+^) and endothelial progenitor cells (EPCs) (CD34^+^/VEGR‐2^+^/CD133^+^) 10. Although fibroblast‐like multipotent cells with proliferative and multidifferentiative properties have been identified in human peripheral blood 11, 12, 13, to date, no evidence about their presence has been reported in L‐PRF products. As the discovery of multipotent stem cells in L‐PRF products could have important implications for the future of regenerative medicine confirming (*i*) the active role of the haemoderivatives in the so‐called *in vivo* guided regeneration and (*ii*) the development of a standardized method to extract autologous stem cells, in this study, a leucocyte–platelet‐concentrated membrane, prepared according to the Caloprisco protocol 10 and called CLP‐MB, has been cultured *in vitro* to characterize the stemness grade of sprouted cells under permissive conditions.

## Materials and methods

### Haemoderivatives

Following the Italian standards of quality assurance, leucocyte‐ and platelet‐rich fibrin membranes (CLP‐MB) were prepared at the Immunohematology and Transfusion Medicine Department, San Martino Hospital of Belluno, Italy. Under Italian ethic committee authorization and informed consent, ten male volunteer donors were submitted to a multicomponent apheresis procedure, and blood samples were processed according to the procedure published by Caloprisco *et al*. 10. In Table 1, haematologic values of blood samples and blood derivatives are reported.

**Table 1:** Haematologic values of blood samples before apheresis (pre‐AP) and at final phase of concentrated leucocyte/platelet membrane (CPL‐MB)

Patient	Phase	RBC (×10^6^/μl)	PLT (×10^3^/μl)	WBC (×10^3^/μl)	NE (×10^3^/μl)	LY (×10^3^/μl)	MO (×10^3^/μl)
1	pre‐AP **CLP‐MB**	5.23 **0.58**	183 **1452**	6.58 **22.40**	4.70 **4.85**	1.10 **9.22**	0.60 **10.17**
2	pre‐AP **CLP‐MB**	4.46 0.37	249 1511	6.27 23.20	4.40 2.80	1.40 16.00	0.30 3.60
3	**pre‐AP** **CLP‐MB**	5.53 **0.37**	184 **1464**	4.36 **17.60**	2.70 **0.80**	1.30 **12.80**	0.30 **4.00**
4	**pre‐AP** **CLP‐MB**	5.40 **0.39**	185 **1700**	6.38 **30.00**	3.20 **0.80**	1.90 **21.20**	0.60 **8.00**
5	pre‐AP **CLP‐MB**	4.18 **0.30**	206 **1800**	4.66 **18.00**	2.90 **1.20**	1.10 **12.40**	0.30 **4.00**
6	pre‐AP **CLP‐MB**	4.96 **0.53**	171 **1801**	4.26 **29.40**	2.30 **2.70**	1.50 **20.30**	0.30 **6.20**
7	pre‐AP **CLP‐MB**	4.87 **1.06**	153 1502	3.47 30.80	1.60 1.60	1.40 22.30	0.40 6.90
8	pre‐AP **CLP‐MB**	5.13 **0.55**	188 **1808**	3.12 **21.00**	1.60 **1.20**	1.00 **13.30**	0.40 **6.50**
9	pre‐AP **CLP‐MB**	5.33 **0.64**	234 **1508**	4.04 **25.20**	1.60 **3.20**	1.6 **15.40**	0.50 **6.60**
10	pre‐AP **CLP‐MB**	4.33 **0.28**	199 **1794**	4.58 **23.25**	2.10 **0.60**	1.80 **17.10**	0.50 **5.55**

RBC: red blood cells; PTL: platelets; WBC: total leucocytes; NE: neutrophils; LY: lymphocytes; MO: monocytes. The representative data of RBC, PLT and leucocytes from CPL‐MB are reported in bold.

### Isolation of fibroblast stem‐like cells

Rounded patches of CPL‐MB were prepared and seeded in polystyrene culture dishes (BD, Franklin Lakes, NJ, USA) preconditioned with foetal bovine serum (Invitrogen‐Thermo Fisher Scientific, Waltham, MA, USA). Thus, samples were maintained at 37°C, 95% humidity and 5% CO_2_, in proliferation culture medium [Alpha‐modified Eagle's medium (α‐MEM) without nucleosides, 50% foetal bovine serum, 1% antibiotic solution, 1% Glutamax (all from Invitrogen Thermo Fisher Scientific, Inc)]. When cell sprouting was observed, CPL‐MB patches were discarded, and culture medium was changed with fresh one every 2 days. Cell expansion was performed for 21 days before detecting fibroblastoid cells (CPL‐CMCs) with proliferative potential. At 80% confluence, CPL‐CMC cells were detached using 0.02% EDTA/0.25% trypsin solution, and subcultures were seeded (5 × 10^3^ cells/cm^2^) in proliferation culture medium containing 16.5% FBS. In alternative to commercial FBS, autologous serum has been suggested to be used in the perspective of clinical use of CPL‐CMCs. During cell isolation and expansion phases, the samples were daily observed by optical microscope DM/IL (Leica, Wetzlar, Germany), and pictures were taken with Nikon Digital Sight Ds‐SMCc camera (Nikon Corporation, Tokyo, Japan). In order to correlate the activation of circulating multipotent cells to the inflammatory environment promoted by haemoderivatives, the expression of TNFα, IL‐10, Wnt3a, TGFβ1, CD206 was investigated by Western blot in cells sprouted from CPL‐MB. In parallel, the expression pattern of CPL‐MB was used as a reference.

### Proteomic analysis of CPL‐derived adherent cells

Using antibodies reported in Table 2, Western blot analysis was performed on total protein extract of CLP membranes and cells isolated from early (inflammatory cells) and late (CLP‐CMCs) sprouted populations. The protein extraction was carried out using a RIPA buffer containing 0.25% TWEEN^®^20 (Sigma‐Aldrich, St. Louis, MO, USA). After quantification using BCA Protein Assay Reagent Kit (Thermo Fisher Scientific, Inc.), 20 μg of total protein extracts from each sample was separated by reducing SDS‐PAGE (Bio‐Rad Laboratories Inc., Hercules, CA, USA) and then electrophoretically transferred to 0.45‐μm nitrocellulose membrane (Immunological Sciences, Rome, Italy). The immunoblot was performed by incubating samples overnight at 4°C with primary antibodies against CD206, TGFβ1, Wnt3a, IL‐10, TNFα (Table 2). After washing with 0.25% TWEEN^®^20 in PBS, the membranes were treated for 1 h with peroxidase‐conjugated secondary goat antimouse and anti‐rabbit antibodies (Immunological Sciences) and then developed using enhanced chemiluminescence substrate (Immunological Sciences). The immunoreactive sites were visualized using VersaDoc Imaging System (Bio‐Rad Laboratories Inc.). The protein expression level was normalized to glyceraldehyde 3‐phosphate dehydrogenase/GAPDH housekeeping protein (EMD Millipore, Billerica, MA, USA) and quantified by ImageLab processing software (Bio‐Rad Laboratories Inc.). Data from three independent experiments were reported as a ratio within the target protein and relative housekeeping protein expression.

**Table 2:** Antibodies used for flow cytometry analysis, Western blot and immunofluorescence

Primary antibodies	Manufacturing company
FITC mouse anti‐human CD11c	BD Biosciences
APC mouse anti‐human CD13	BD Biosciences
PE mouse anti‐human CD14	Santa Cruz Biotecnology, Inc
FITC mouse anti‐human CD29	Santa Cruz Biotecnology, Inc
PE mouse anti‐human CD31	BD Biosciences
PE‐Cy5 mouse anti‐human CD33	BD Biosciences
PE‐Cy7 mouse anti‐human CD34	BD Biosciences
Rabbit anti‐human CD38	Santa Cruz Biotecnology, Inc
PE mouse anti‐human CD40	BD Biosciences
PE mouse anti‐human CD44	Santa Cruz Biotecnology, Inc
PE mouse anti‐human CD45	Santa Cruz Biotecnology, Inc
FITC mouse anti‐human CD49f	ImmunoTools
PE mouse anti‐human CD73	BioLegend, Inc
PE‐Cy5 mouse anti‐human CD80	BD Biosciences
APC mouse anti‐human CD86	BD Biosciences
FITC mouse anti‐human CD90	Santa Cruz Biotecnology, Inc
Mouse anti‐human CD103	Santa Cruz Biotecnology, Inc
PE mouse anti‐human CD105	Santa Cruz Biotecnology, Inc
FITC mouse anti‐human CD106	Acris Antibodies GmbH
APC mouse anti‐human CD133/1	Miltenyi Biotec
PE mouse anti‐human CD133/2	Miltenyi Biotec
FITC mouse anti‐human CD146	Santa Cruz Biotecnology, Inc
FITC mouse anti‐human CD166	Santa Cruz Biotecnology, Inc
Rabbit anti‐human CD206	Santa Cruz Biotecnology, Inc
PE mouse anti‐human PDGFRβ	BD Biosciences
FITC mouse anti‐human VEGFR2	R&D Systems, Inc.
Rabbit anti‐human FGFR1	Santa Cruz Biotecnology, Inc
APC mouse anti‐human FGFR2	R&D Systems, Inc.
Rabbit anti‐human EGFR	Santa Cruz Biotecnology, Inc
FITC mouse anti‐human NG2	Santa Cruz Biotecnology, Inc
Rabbit anti‐human VE‐cadherin	Santa Cruz Biotecnology, Inc
Mouse anti‐human αSMA	EMD Millipore
Mouse anti‐human vimentin	Santa Cruz Biotecnology, Inc
Rabbit anti‐human vWF	Abcam
Mouse anti‐human FVIII	Abcam
Rabbit anti‐human TGFβ1	Santa Cruz Biotecnology, Inc
Rabbit anti‐human Wnt3a	Immunological Sciences
Rabbit anti‐human IL‐10	Immunological Sciences
Rabbit Anti‐human TNFα	Immunological Sciences
PE mouse anti‐human CXCR4	Santa Cruz Biotecnology, Inc
Rabbit anti‐human Frizzled 1	Acris Antibodies GmbH
PE mouse anti‐human Frizzled 2	Santa Cruz Biotecnology, Inc
Goat anti‐human Frizzled 3	Santa Cruz Biotecnology, Inc
Goat anti‐human Frizzled 9	Santa Cruz Biotecnology, Inc
PE mouse anti‐human SEEA4	BD Biosciences
Alexa Fluor^®^ 488 mouse anti‐human TLR4	Bioss Antibodies
Rabbit anti‐human SIRPα	eBiosciences
FITC mouse anti‐human GR‐1/Ly6G	BD Biosciences
PE‐Cy7 mouse anti‐human HLA‐ABC	BD Biosciences
PE‐Cy5 mouse anti‐human HLA DR	BD Biosciences

**Secondary antibodies**	
PE goat antimouse	Santa Cruz Biotecnology, Inc
PE goat anti‐rabbit	Santa Cruz Biotecnology, Inc
PE donkey anti‐goat	Santa Cruz Biotecnology, Inc
Alexa Fluor^®^ 488 antimouse	EMD Millipore

**Isotype controls**	
FITC Isotype Control	Santa Cruz Biotechnology, Inc; BD Biosciences; ImmunoTools; Acris Antibodies GmbH; R&D Systems, Inc
PE Isotype Control	Santa Cruz Biotechnology, Inc; BD Biosciences; BioLegend, Inc; Miltenyi Biotec
PE‐Cy5 Isotype Control	BD Biosciences
PE‐Cy7 Isotype Control	BD Biosciences
APC Isotype Control	R&D Systems, Inc.; Miltenyi Biotec.
Alexa Fluor^®^ 488 Isotype Control	Bioss Antibodies

### Proliferation capacity of CPL‐CMCs

The proliferation rate of CPL‐CMCs was evaluated culturing for 16 consecutive passages and seeding in triplicate each generation at a density of 5 × 10^3^ cells/cm^2^. After 24 hrs, the cells were detached using EDTA/trypsin solution and counted with a hemocytometer. The average number of cells and standard deviation (SD) for each passage was used to define the doubling population time (DPT).

### Stemness gene profile

The expression of OCT4, NANOG, SOX2, KLF4, NOTCH, STAT3 and REX1 was investigated by quantitative PCR (qPCR) in subcultures evolving from 4th generation to 20th generations, using oligonucleotides (Thermo Fisher Scientific, Inc.) listed in Table 3. Reverse transcription and amplification reaction were carried out using SensiFAST™ SYBR^®^ One‐Step kit (Bioline Inc., London, UK) and AriaMx Real‐time PCR System (Agilent Technology, Santa Clara, CA, USA). For statistical significance of data, three independent analyses of each target per subpopulation were performed preparing multiple technical replicates. The hypoxanthine phosphoribosyltransferase 1/HPRT1 housekeeping gene was considered as control. For quantification of gene expression level, the comparative CT method (2^−ΔCt^) was used.

**Table 3:** Oligonucleotides used for RT‐PCR and qPCR analysis (F = forward; R = reverse)

Genes	Primer sequences	Accession	Amplicon length
TGFA	F: CACACTCAGTTCTGCTTCCA R: GTGATGGCCTGCTTCTTCT	NM_003236.3	151 bp
TGFB1	F: CGTGGAGCTGTACCAGAAATAC R: CTAAGGCGAAAGCCCTCAAT	NM_000660.6	158 bp
LIF	F: TCTGCACTGGAAACATGGG R: CTGATCTGGTTCATGAGGTTGT	NM_002309.4	104 bp
IL1A	F: AGTGAGACCAACCTCCTCTT R: ACACCCAGTAGTCTTGCTTTG	NM_000575.4	108 bp
IL1B	F: ATGGACAAGCTGAGGAAGATG R: CCCATGTGTCGAAGAAGATAGG	NM_000576.2	114 bp
IL4	F: CACCGAGTTGACCGTAACA R: CTTCCATGGTGGCTGTAGAA	NM_000589.3	138 bp
IL6	F: GAGCTGTGCAGATGAGTACAA R: GGACTGCAGGAACTCCTTAAA	NM_000600.4	190 bp
IL10	F: GCTGGAGGACTTTAAGGGTTAC R: GATGTCTGGGTCTTGGTTCTC	NM_000572.2	105 bp
IL12	F: ATTCCAGAGAGACCTCTTTCATAAC R: CTTGAACTCCACCTGGTACATC	NM_000882.3	124 bp
TNFA	F: CCAGGGACCTCTCTCTAATCA R: TCAGCTTGAGGGTTTGCTAC	FJ795028.1	106 bp
TNFR1	F: GGACAGGGAGAAGAGAGATAGT R: TGGACAGTCATTGTACAAGTAGG	AH003016.2	115 bp
TNFR2	F: TGCATCGTGAACGTCTGTAG R: GGAATCTGTGTCTCCCATTGT	NM_001066.2	84 bp
REX1	F: GAGATGGAGTAAGGAGGGAGAT R: ATGGACAAGCTGAGGAAGATG	NM_174900.3	105 bp
SOX2	F: ATTCCAGAGAGACCTCTTTCATAAC R: TACCTTTCTGCCCGTGAAGTGGAT	NM_003106.3	191 bp
STAT3	F: AAAGACAGCTACGTGGGTGACGAA R: AGAACCTGCAGGAGGCAGAAGAAT	NM_213662.1	175 bp
NOTCH	F: AGGATCACACAGGTGGCCCATATT R: AGCTAAAGCAGCAGCAAACTTCGG	NM_017617.3	112 bp
OCT4	F: TCGAGGAATTGCTCAAAGTGCTGG R: ACACCAACGGCTGGAAGCTAAATC	NM_002701.4	102 bp
KLF4	F: GAAGATGCGCAGCAGCGAGAATTT R: ACTTTCTCCTGTCCGTCATTGGCT	NM_004235.4	106 bp
NANOG	F: AGAATATGCACCAGGCCGAAGAGT R: AGCTAAGAGTCTTTGGTGCTGGCT	NM_024865.2	106 bp
TUBβ3	F: ACAACGAGGCCTCTTCTCACAAGT R: ATACTCCTCACGCACCTTGCTGAT	NM_006086.3	225 bp
vWF	F: ACTCAGTGCATTGGTGAGGATGGA R: TCGGACACACTCATTGATGAGGCA	NM_000552.4	842 bp
CD31	F: ACTGGACAAGAAAGAGGCCATCCA R: TCCTTCTGGATGGTGAAGTTGGCT	NM_000442.4	677 bp
HPRT1	F: ATGGACAGGACTGAACGTCTTGCT R: TTGAGCACACAGAGGGCTACAATG	NM_000194.2	79 bp

### Immunophenotyping of CPL‐CMCs

Using anti‐human antibodies reported in Table 2, subcultures from 4th to 20th generations were analysed by flow cytometry (FCM) for the expression of typical markers related to stemness, lineage commitment, cell–ECM interactions and enzyme/signalling molecules. Flow cytometry analysis was performed with FACSCanto II Flow cytometer (BD Biosciences, CA, USA) and FACS Diva software (BD). Data were reported as mean percentage of positive cells and relative mean fluorescence intensity (MFI) calculated on *n* = 3 replicas of each sample for all target markers. Samples treated with only secondary antibodies or isotype control antibodies (Table 2) were prepared as references.

### Differentiative plasticity of CPL‐CMCs

CLP‐CMCs were seeded at 1.5 × 10^4^ cells/cm^2^ and induced to differentiate under the conditions described below. In parallel, cultures in proliferation medium were prepared as controls. After 7 and 14 days, the analysis by cytochemistry, immunofluorescence, PCR, WB and FCM was performed to confirm the lineage‐specific differentiation. In all experiments, resting cells were used as reference. Antibodies and oligonucleotides are reported in Table 2 and Table 3, respectively. For gene expression analysis, the housekeeping HPRT1 was considered for normalization of data.

### Adipogenic induction

The stimulation was performed with DMEM high‐glucose medium (Sigma‐Aldrich) supplemented with 10% FBS (Invitrogen‐Thermo Fisher Scientific, Inc), 1% antibiotic solution (Sigma‐Aldrich) and adipogenic supplements (1 mM dexamethasone, 0.5 mM 3‐isobutyl‐1‐methylxanthine, 10 mg/ml insulin, 60 mM indomethacin) (all from Sigma‐Aldrich). After 3, 7 and 14 days from induction, the adipogenic commitment was evaluated detecting the expression of perilipin/PLIN1 and leptin/LEP genes by qPCR. In parallel, samples were fixed with 10% formalin solution (Sigma‐Aldrich) and stained with Oil Red O solution (5 mg/ml in isopropanol) (Sigma‐Aldrich) to verify the presence of cytoplasmic lipid droplets. Nuclei were counterstained with haematoxylin (Sigma‐Aldrich) according to the standard procedure.

### Myogenic induction

Subconfluent (~90%) CLP‐CMCs were grown in standard medium supplemented with 100 ng/ml IGF (ImmunoTools, Friesoythe, Germany) and 200 μM ascorbic acid (Sigma‐Aldrich Co.). At different time‐points (3, 7, 14 days), myogenic differentiation was verified by qPCR investigating the expression of genes related to early (myogenic differentiation 1/MYOD1), intermediate (myogenin/MYOG) and late (tropomyosin 1/TPM1) differentiation phases. At 14 days from induction, the expression of vimentin was evaluated by immunofluorescence to detect the formation of syncytium‐like structures.

### Neurogenic induction

Neurogenic differentiation was induced in subconfluent (~60%) cells using DMEM/F‐12 medium (Thermo Fisher Scientific, Inc.) supplemented with 2% FBS, 50 U/ml penicillin, 50 μg/ml streptomycin and 0.1% dimethyl sulphoxide (DMSO) (all from Sigma‐Aldrich). After 3 and 7 days, the samples were submitted to the analysis of brain‐derived neurotrophic factor/BDNF, nerve growth factor/NGF, tubulin beta 3 class III/TUBB3 and synaptophysin/SYP by qPCR.

### Endothelial induction

CLP‐CMCs (2x10^4^cells/cm^2^) were seeded on 24‐well plates coated with 0.5 ml of Matrigel (diluted 1:10 in standard medium) (BD Bioscience) and cultured in a humid atmosphere with 5% CO_2_. At 3 and 7 days from induction, the gene expression of platelet endothelial cell adhesion molecule/CD31 was analysed by one‐step reverse transcriptase–PCR (Qiagen, Hilden, Germany). In parallel, we evaluated the formation of capillary‐like structures by optical microscopy using an inverted microscope Motic AE2000 (Motic^®^, Wetzlar, Germany) equipped with Nikon DS‐L1 camera (Nikon, Düsseldorf, Germany), and the expression of blood‐clotting protein factor VIII/FVIII by immunofluorescence. Finally, to better explore the endothelial potential of CLP‐CMC cells, extracellular vesicles/exosomes (EVs/exs) were isolated using Cell Culture‐Nanovesicles kit (Biofield Innovation Srl, Padova, Italy) from conditioned media according to the manufacturer's protocol. After labelling with PKH26 Red Fluorescent Cell Linker Kit for General Cell Membrane Labeling (Sigma‐Aldrich), all samples were characterized by FCM for size, using as reference polystyrene beads supplied in Flow Cytometry Size Calibration Kit (Molecular Probes, Inc, Eugene, OR), and expression of CD9 or CD63 by indirect staining, according to the Pospichalova protocol 14. Moreover, we analysed by WB the expression of tetraspanin family protein/CD9, FVIII, Wnt3a ligand. Extracellular vesicles/exosomes from resting cells were considered as reference. For excluding cells from the analysis, cis‐Golgi marker/GM‐130 was considered as staining control. All antibodies used are listed in Table 2.

## Statistical analysis

It was performed with paired Student's *t*‐test, and results were considered significant when *P *<* *0.05.

## Results

### Isolation and growth of human CPL‐CMC cells

**Figure 1:**
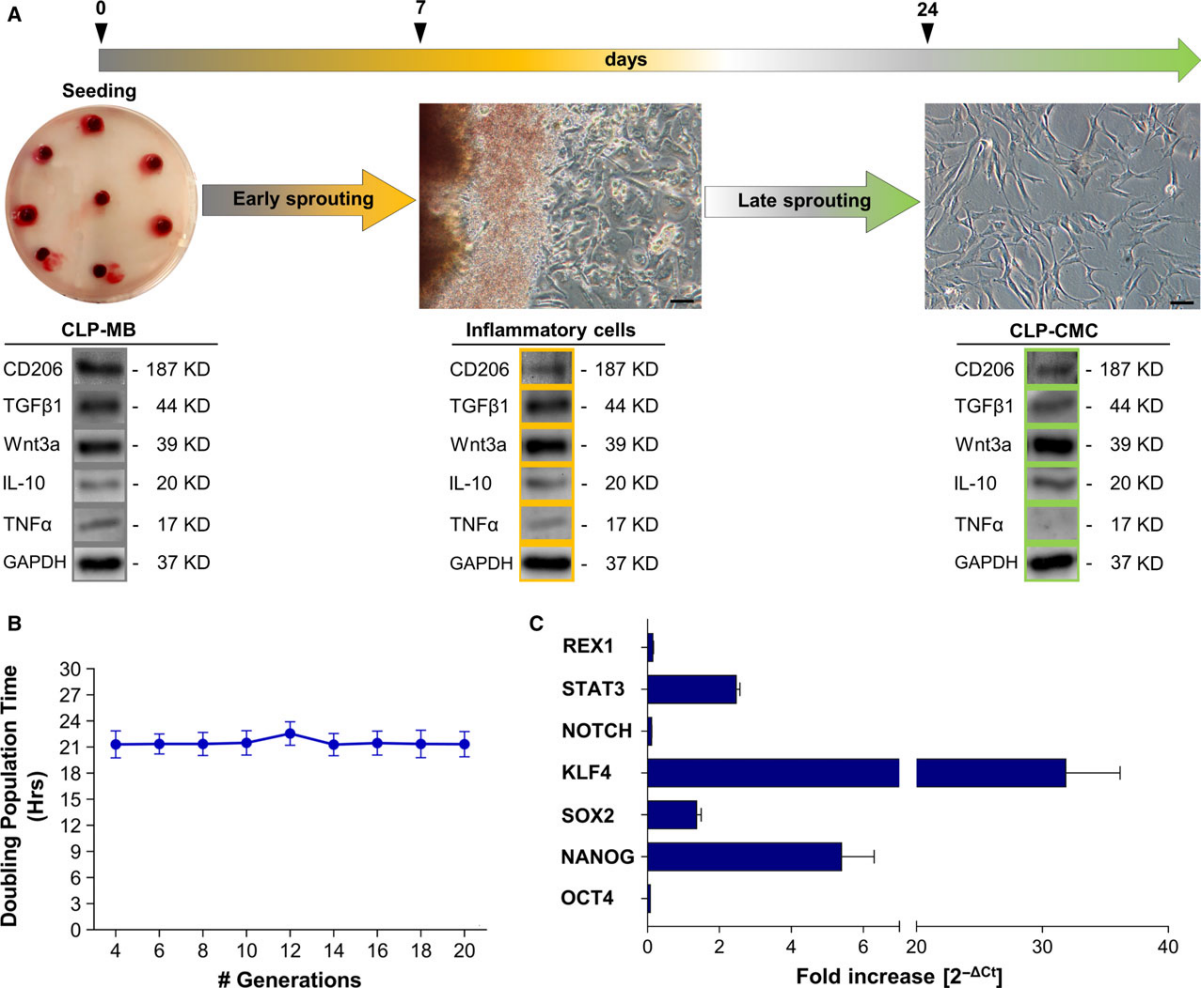
Compared to other blood‐derived stem cell populations, CLP‐CMCs have a distinctive stemness signature. Morphological study and stemness characterization of human CLP‐CMCs. (**A**) Optical microscopy image of CLP‐MB and CLP‐CMC sprouted cells at early and late‐phases during 21 days of *in vitro* culturing. Scale bar: 25 μm. (**B**) Calculation of doubling population time (DPT) over a total of 16 divisions. (**C**) Gene expression analysis of pluripotency markers by quantitative PCR in cells grown in proliferative medium. The comparative CT method (2^−ΔCt^ ± S.D) was used to quantify the gene expression level. *HPRT* was considered as housekeeping gene.

The study included 10 male volunteers under therapy with haemoderivatives for impaired wound healing. After 21 days from seeding, all samples showed an active cell sprouting with spindle‐ or flat‐like shaped cells at early‐phase and cells with fibroblastic morphology at late‐phase (Fig. 1A). Accordingly, a different expression pattern of the inflammatory cytokine TNFα and the protective molecule IL‐10 was observed (Fig. 1A), suggesting a possible correlation among *in vivo* regeneration following the implantation of CLP‐MB and the *in vitro* development of cells with anti‐inflammatory functionality, proliferative activity and high grade of stemness. In particular, CPL‐CMC subcultures from 4th to 20th generation demonstrated a doubling population time of 21 ± 1.85 hrs, which was significantly shorter than that of other multipotent cells 12, 13 isolated from human peripheral blood (Fig. 1B). During *in vitro* short and prolonged expansion, a high positive expression of transcription factors NANOG, SOX2, KLF4, STAT3 was detected (Fig. 1C), suggesting a high stemness grade of CPL‐CMCs. In parallel, normal karyotype of 46 chromosomes with no aneuploidy, tetraploidy or other visible abnormalities was verified (data not shown).

### Multipotency of CPL‐CMCs

**Figure 2:**
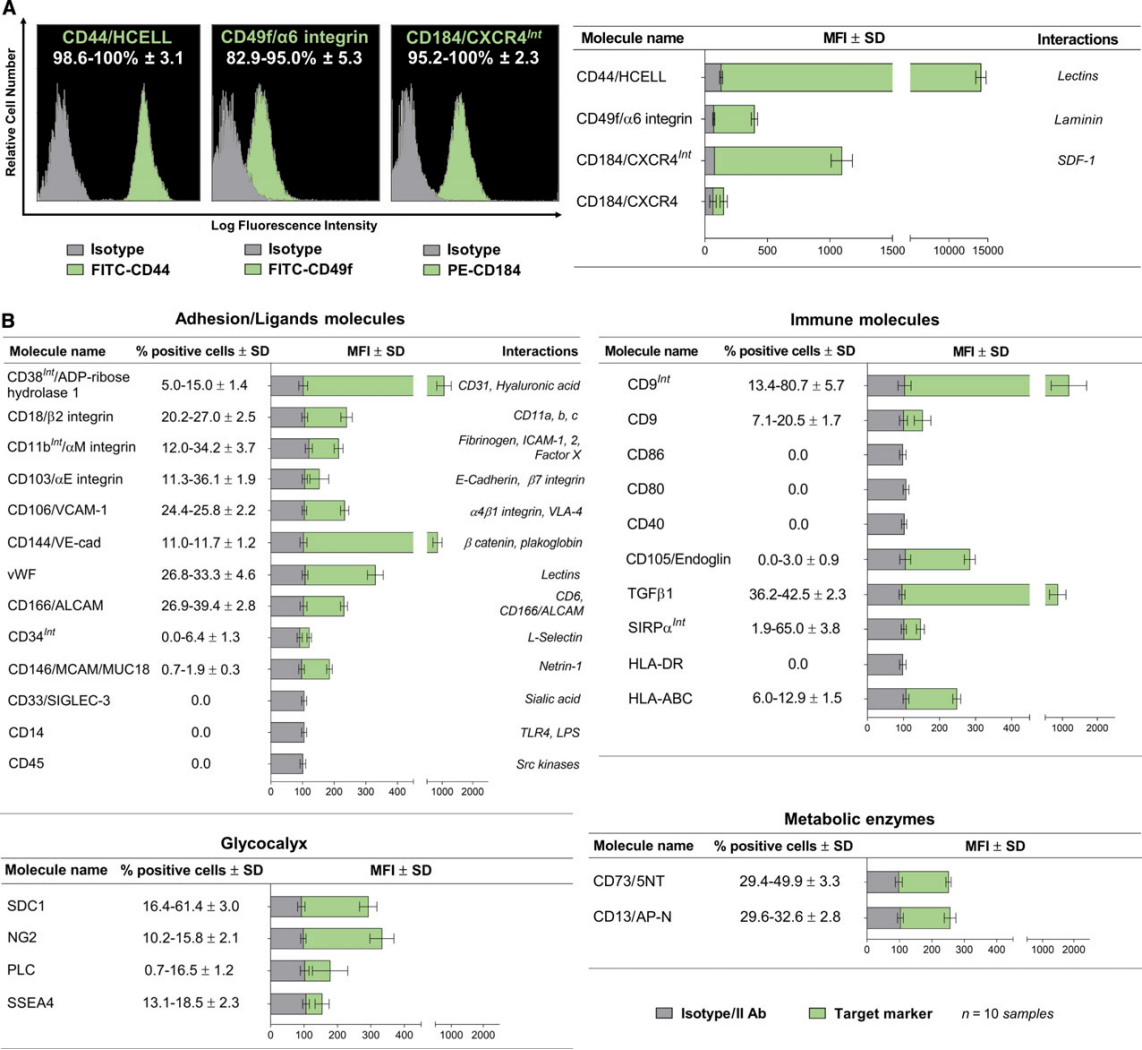
CLP‐CMCs proliferate *in vitro* without reaching replicative senescence and growth arrest. FCM characterization of CLP‐CMC subcultures under proliferative conditions. Data are reported as mean percentage of positive cells and relative mean fluorescence intensity (MFI) calculated on *n *=* *3 replicas of each sample for all target markers. Samples treated with only secondary antibodies or isotype control antibodies were used as references.

By FACS analysis, the immunophenotypic profile of CMC was determined (Fig. 2). Interestingly, all populations extracted from CPL membranes showed an almost homogenous expression of CD44/HCELL, CD49f and CD184/CXCR4 (Fig. 2A) that are markers related to bone marrow derivation 15, multipotency 16 and migratory potentialities 17. As expected, several markers typically expressed in multipotent stem cells or mediating transendothelial migration, angiogenic potentiality, cell–matrix and cell–cell interactions, and finally immune properties were detected in CPL‐CMCs. They included CD13, CD73, CD105, SSEA4, NG2 as stem cell markers; CD106, CD144, CD146, CD166, von Willebrand factor/vWF as endothelial stem/progenitor phenotype cues; and CD11b, CD18, CD103 as adhesion molecules (Fig. 2B). Glycolipids 18, such as NG2, and heparan sulphate proteoglycans 19, such as syndecan‐1/SDC1 20, 21 and perlecan/PLC 21, are critical environmental regulators of haematopoietic and mesenchymal stem cell niches. As reported in Fig. 2B, CPL‐CMC cells showed to express SDC1 and PLC that, together with CD34 and CD38, were assumed as indicative of both adhesive properties to endothelium and possible derivation from bone marrow. Other markers such as CD14, CD29, CD31, CD45, CD90, CD117, CD133, PDGFRβ were not detected (Fig. 2B). Moreover, CPL‐CMCs demonstrated by FCM to possess immune properties and engraftment potential expressing tetraspanin CD9 22, TGFβ 23 and SIRPα 24 (Fig. 2B). For CD9, a role in migration, adhesion and homing is also considered 25, 26. The null expression of HLA Class II and the low level of HLA Class I confirmed the potentialities of CPL‐CMCs for both allogeneic and autologous therapies 27. Taken together, our collected data demonstrated distinctive immunophenotypic properties of CPL‐CMCs as compared to other blood‐derived stem cell populations.

### Responsiveness of CPL‐CMC cells to environmental stimuli

**Figure 3:**
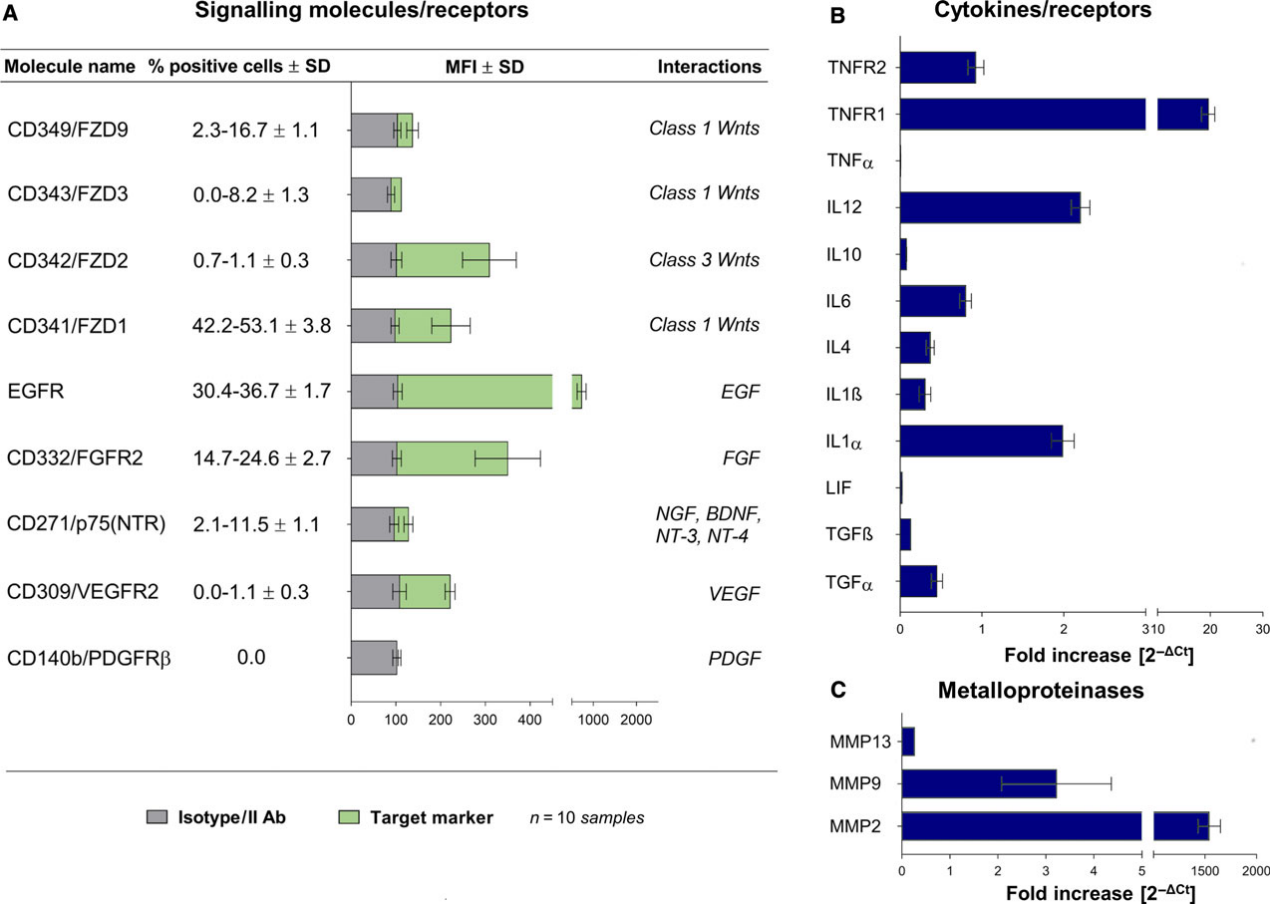
High responsivity to Wnt signals, growth factors, neurotrophins and potential migratory activity are observed in CLP‐CMCs. (**A**) Evaluation of CLP‐CMC responsivity to environmental stimuli by FCM. (**B**) Gene expression analysis of immune properties and matrix remodelling enzymes by qPCR.

As reported in Fig. 3A, CPL‐CMCs showed by WB the expression of numerous growth factors, including EGF, FGF, neurotrophins and Wnt ligands. The synthesis of pro‐ and anti‐inflammatory cytokines (Fig. 3B), receptors of TNFα (Fig. 3B) and matrix remodelling enzymes such as MMP‐2, MMP‐9 and MM‐P13 (Fig. 3C) was detected at mRNA level.

### Differentiative potentialities of CPL‐CMCs

**Figure 4:**
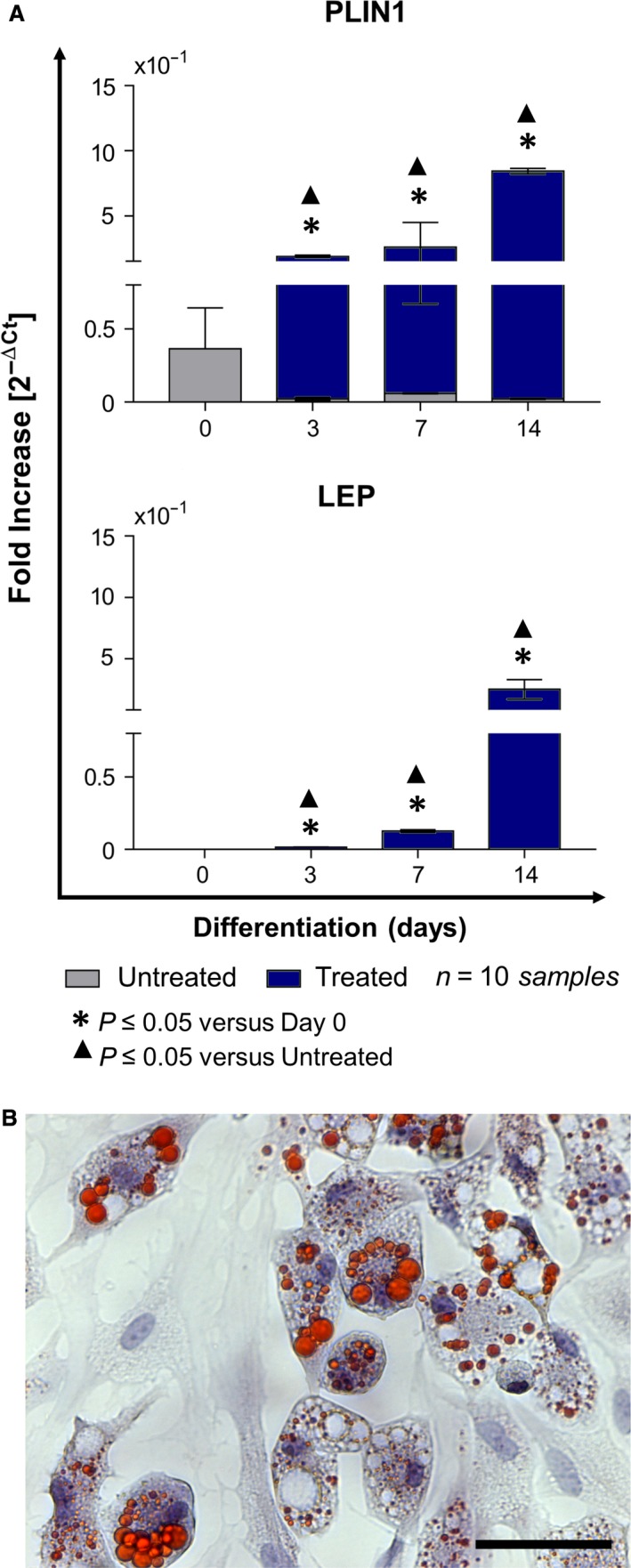
Under permissive *in vitro* conditions, CLP‐CMCs acquire adipocyte‐like phenotype. (**A**) Gene expression analysis of perilipin/PLIN1 and leptin/LEP. (**B**) Detection of cytoplasmic lipid droplets by Oil Red O staining. Nuclei were counterstained with haematoxylin. Scale bar: 25 μm.

**Figure 5:**
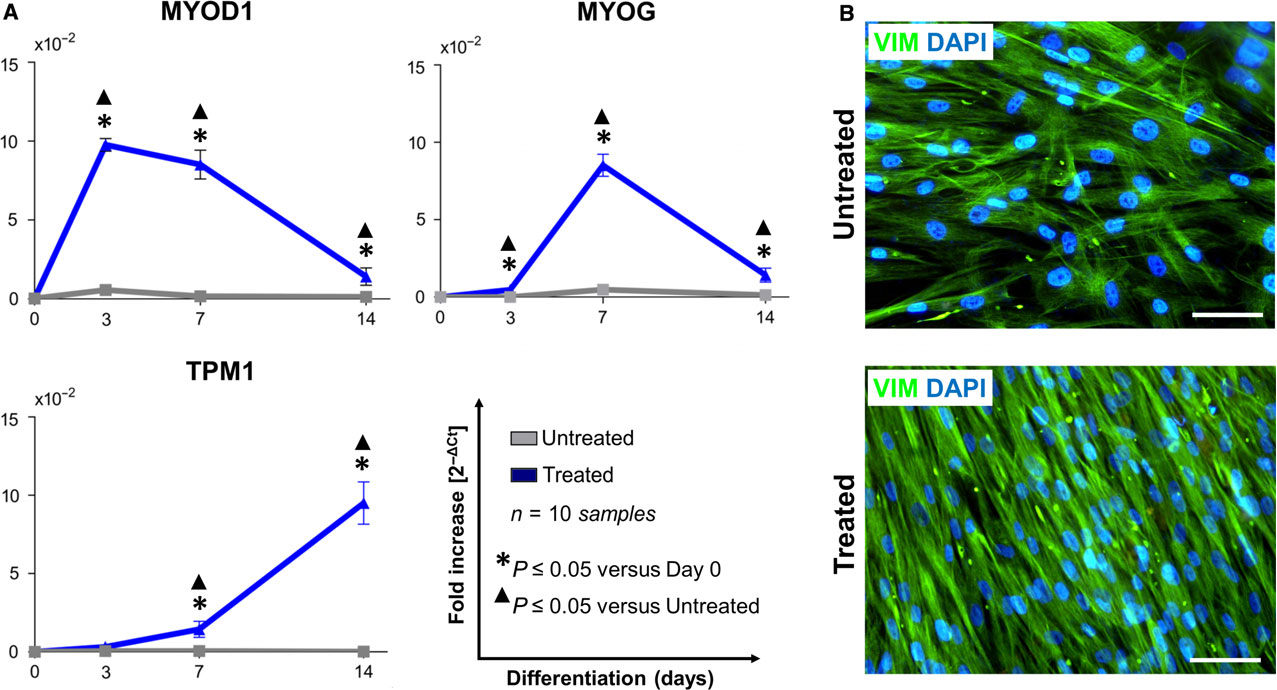
CLP‐CMCs have a potential to undergo myogenic‐like commitment. (**A**) qPCR analysis of MYOD1, myogenin/MYOG) and tropomyosin/TPM1. (**B**) Immunofluorescence detection of vimentin. Nuclei were counterstained with Fluoro‐Gel II solution containing DAPI. Scale bar: 25 μm.

**Figure 6:**
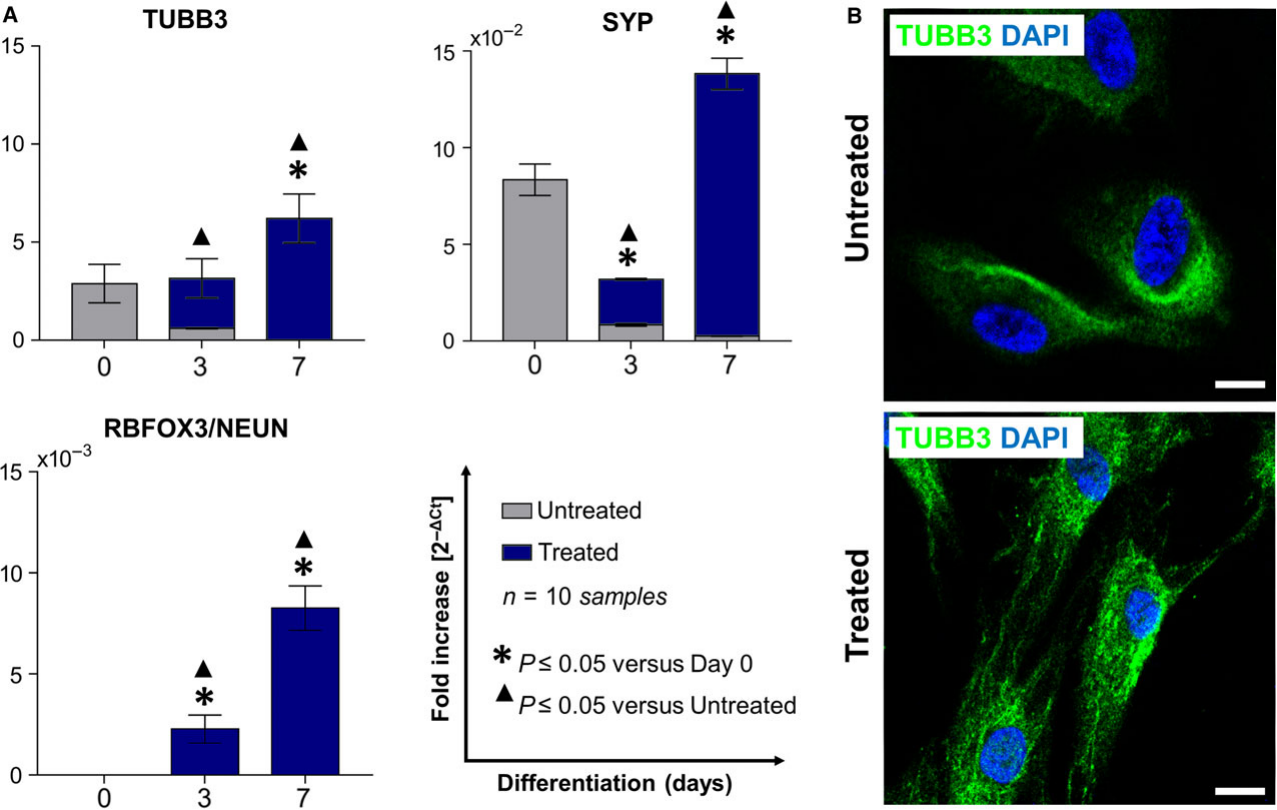
Neurogenic differentiative ability of CLP‐CMCs is demonstrated *in vitro*. (**A**) qPCR analysis of ß‐tubulin isotype III/TUBB3, synaptophysin/SYP and neuronal nuclear antigen RBFOX3/NEUN). (**B**) Immunofluorescence detection of TUBB3. Nuclei were counterstained with Fluoro‐Gel II solution containing DAPI. Scale bar: 10 μm.

**Figure 7:**
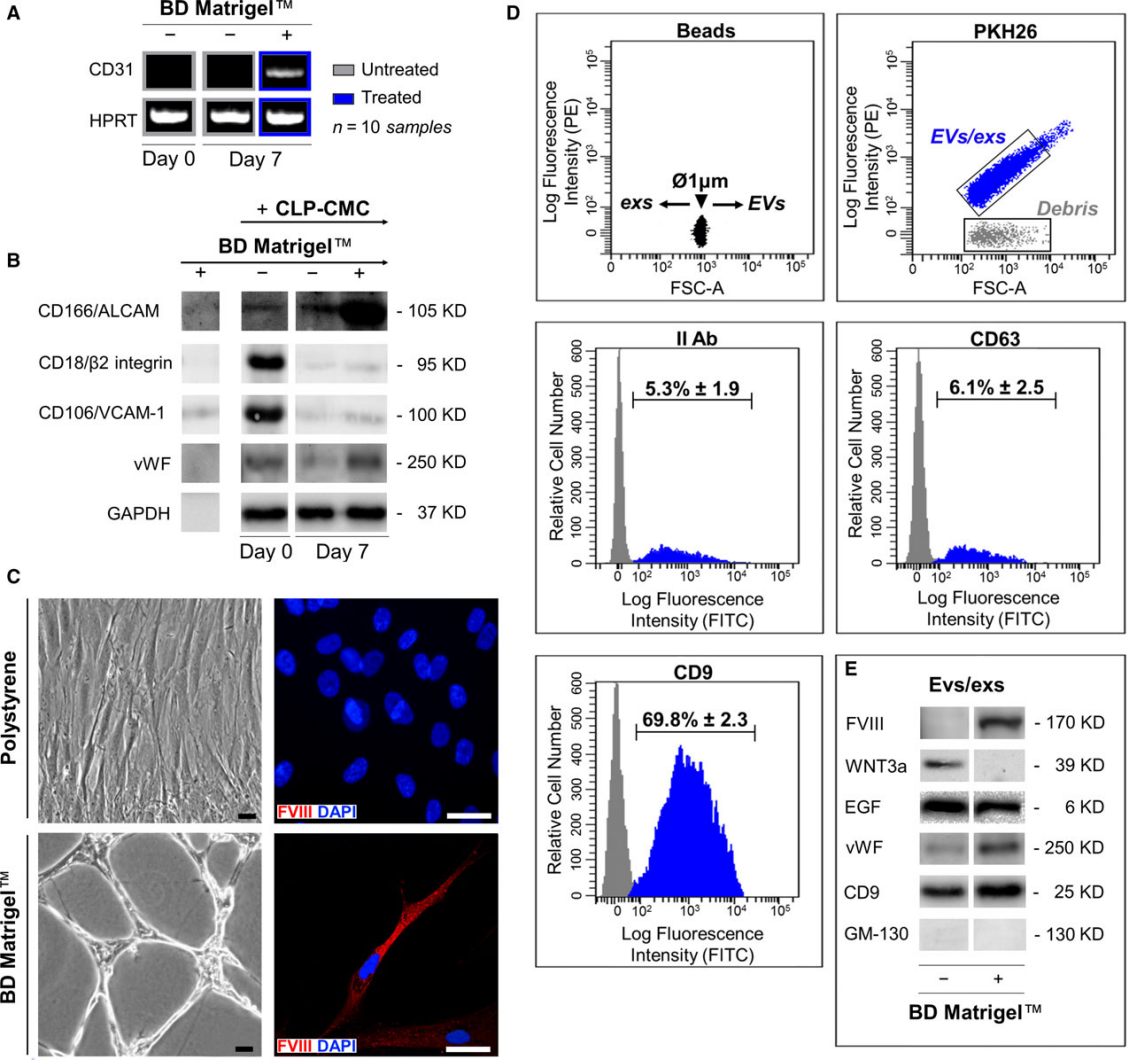
In comparison with resting cells (−), CLP‐CMCs respond to the stimulation with BD Matrigel^TM^ (+) acquiring endothelial‐like phenotype. (**A**) Analysis of CD31 gene by *one‐step* RT‐PCR. (**B**) WB analysis of vascular adhesion molecules (CD18, CD106, CD166), vWF and GAPDH housekeeping protein. (**C**) Optical microscopy (*left side)* and immunofluorescence (*right side*) detecting FVIII in cells counterstained with DAPI. Scale bar: 25 μm. (**D**) FCM characterization of PKH26‐tagged extracellular vesicles/exosomes [isolated from the conditioned culture media of CLP‐CMCs stimulated with BD Matrigel^TM^ (+) or resting cells (−). The vesicles were discriminated by size, using polystyrene beads as reference, and by expression of characteristic markers, CD9 and CD63. (**E**) WB analysis of FVIII, WNT3a, EGF, vWF, CD9 in extracellular vesicles/exosomes isolated from the conditioned culture media of CLP‐CMCs stimulated with BD Matrigel^TM^ (+) or resting cells (−). To verify the absence of cells, the expression of cis‐Golgi marker/GM‐130 was considered as negative control.

After specific differentiation induction, the plasticity of CMCs towards adipogenic, myogenic and neurogenic lineages was demonstrated evaluating the gene expression of lineage‐specific markers (Fig. 4A, Fig. 5A, Fig. 6A), the accumulation of lipid droplets (Fig. 4B), the acquisition of cell‐orientated distribution (Fig. 5B) and the assembling of cytoskeleton components (Fig. 6B). CPL‐CMCs displayed the gene expression of CD31 (Fig. 7A), an increased protein expression of CD166 and vWF (Fig. 7B) and the formation of capillary‐like network structures (Fig. 7C). The acquired endothelial‐like phenotype was furtherly confirmed by the protein expression of FVIII together with EGF and vWF, in CD9‐tagged mixed population of extracellular vesicles and exosomes (Fig. 7D). In parallel, resting cells cultured on polystyrene culture dishes showed elongated morphology at maximum confluence (Fig. 7C) and expressed Wnt3a by Evs/exs (Fig. 7D).

## Discussion

Offering the cellular, physical and chemical cues implicated in haemostatic response and tissue restoration, the haemoderivatives show great potentials for regenerative medicine 28.

Opening new perspectives in autologous stem cell research, L‐PRF products prepared according to Caloprisco's method 10 have been found to deliver stem cell‐like cells with unique phenotypic features, functionality and differentiative potentialities in comparison with endothelial/haematopoietic progenitors 29, 30, mesenchymal stem cells (MSCs) 27, 31, 32, embryonic‐like stem cells 11, 33, 34, 35, 36 and circulating multipotent cells 12, 13, 25, 37. Isolated by minimal *in vitro* manipulation of CPL membranes, CMC cells showed fibroblast‐like morphology, long‐lasting proliferative activity and high expression of CD44/HCELL, CD49f and CXCR4. The overlapping expression of embryonic (SSEA‐4), mesenchymal (CD13, CD105, CD106, CD73, CD146), haematopoietic (CD38, CD34) and endothelial (CD144, CD166, vWF) stem cell markers highlighted the presence of distinct immature subsets. Moreover, specific immunomodulatory (CD9, SIRPα, pro‐ and anti‐inflammatory cytokines) properties and receptors for Wnt ligands (FZD1/2/3/9), growth factors (EGFR, FGFR2, p75, VEGFR2) and inflammatory stimuli (TNFR1/2) confirmed high environmental responsiveness of CPL‐CMCs. Lacking integrin β1, CD90 and PDGFRβ, CMC cells showed distinct immunophenotype and origin in comparison with circulating multipotent progenitor cells 12, 38 and perivascular multipotent progenitor cells 22, 39. Primed by the interaction with fibrin matrix and P‐selectins on activated platelets 40, 41, the immunophenotypic heterogeneity of CPL‐CMCs has been suggested to reflect a dynamic equilibrium between the acquired responsivity to extracellular signals and the retained self‐renewal potential 42. Based on the expression profile of adhesion molecules (CAMs) and glycolipids/proteoglycans, the physiological and regulatory processes underlying the trafficking of CLP‐CMCs in peripheral blood were defined as similar to those of leucocytes.

Likely haematopoietic stem and progenitor cells, CLP‐CMCs displayed the specialized glycoform of CD44 known as HCELL, suggesting to have a possible haematopoietic origin, bone marrow derivation and transendothelial migration potential. As reported by Sackstein 15, the cell migration from vascular to extravascular compartments develops by two different mechanisms: the canonical multistep process and the so‐called step 2‐bypass pathway. In the canonical pathway, following the initial tethering/rolling contact of blood‐borne cells with endothelium, CXCR4 binds to its cognate ligand CXCL1/SDF‐1, thereby triggering G protein‐coupled VLA‐4 activation, with subsequent firm adhesion and transmigration. In the ‘step 2‐bypass pathway’, the activation of VLA‐4 occurs *via* G protein‐mediated mechanosignaling after HCELL binding to E‐selectin and/or CD44 interaction with endothelial HA. As suggested by the intracellular expression of CXCR4, the extravasation of CPL‐CMCs is likely to progress by the canonical pathway.

The stemness signature of CPL‐CMCs was further confirmed by both the gene expression of the key components of self‐renewal machinery (NANOG, SOX2, KLF4, STAT3) 43, 44, 45 and the almost homogenous expression of CD49f 46, that is known for transducing survival signals, mediating endothelial progenitor cell migration/adhesion and enhancing multipotency through OCT4, SOX2 and NANOG 12, 43. Collectively, our data pointed out that the self‐renewal of CPL‐CMCs could be regulated likely in embryonic stem cells wherein KLF4 connects STAT3 activation with NANOG expression after interacting with SOX2 and OCT4 45.

A growing body of evidence suggests that L‐PRF products, including CPL‐MB, are attractive biomaterials for regenerative medicine due to their ability to promote *in vivo* a guided tissue regeneration by ‘endogenous cell homing’. Our findings add a novel aspect to the complex picture of stem cell research demonstrating that CPL‐MB functions as a reservoir of autologous stem cells that could be isolated under permissive conditions by culturing *ex vivo* the haemoderivatives. Currently, apart from HSC transplantation for haematologic diseases, the clinical experience with somatic stem cell therapy appears promising but still restricted by biological questions about the safety of cell manufacturing, the route of administration, the irreversibility of treatment and the not predictable long‐term survival of engrafted cells 47, 48. Due to these limitations, and considering that, during *in vitro* expansion of multipotent stem cells, the homing molecules could be lost causing a significant reduction in cell migratory efficiency 49, 50, 51, circulating endogenous stem/progenitor cells could represent a valid and alternative source of immature cells for medical autologous applications 52. Responding to a temporally defined sequence of instructive cues mostly derived from platelets, haematopoietic and not haematopoietic cells from CPL membranes shape a co‐ordinated progression from inflammation to regenerative‐like phases, mimicking the response of living tissues to injury 53, 54, 55, 56. One of the key findings of our study was the recognition of CLP‐CMCs as cells expressing striking morphological and immunophenotypic similarities to alternatively activated M2 macrophages 57 and myeloid angiogenic cells (MACs) 58. Showing an elongated/spindle shape and the expression of mannose receptor/CD206, IL‐10, TGFβ1 and Wnt3a 59, 60, CPL‐CMC cells have been suggested to function by dampening inflammatory responses, scavenging cellular debris and promoting angiogenesis. Furthermore, the engagement of CD206 signalling could be also involved in the cell trafficking of CPL‐CMCs through the stimulated production of MMPs (*i.e*. MMP‐2, MMP‐9) and matrix remodelling 61. Indeed, in the early‐phase of *in vitro* culture, CPL membranes showed a significant cell sprouting, thus indirectly suggesting that a proteolytic activity and fibrinolysis were active. The fibrinolytic system, with its main player plasmin, plays a crucial role in cell migration, bioavailability of growth factors and regulation of other protease systems during inflammation and tissue regeneration 28, 62. As the internalization and the degradation of plasminogen activator require the mannose receptor 63, it is likely that CPL‐CMCs could modulate the process of fibrinolysis within CPL membranes for regulating their proliferation, differentiation 62, 64, 65 and migration 61.

Finally, based on their abilities to respond to differentiative stimuli, a broad range of medical applications of CLP‐CMCs could have been suggested, including the treatment of (*i*) blood disorders, such as haemophilia 66; and (*ii*) defects of adipose tissue, skeletal muscle and nervous system 67.

## Conclusion

As a direct evolution of fibrin glue technologies, autologous platelet preparations are a new generation of biomaterials used in regenerative medicine for improving tissue healing. In this study, we demonstrated that the leucocyte‐ and platelet‐rich fibrin product called CPL‐MB functions not only as a reservoir of bioactive factors (PDGF, TGF‐β, VEGF, fibrinogen, fibronectin and vitronectin), useful to recruit stem cells to wound site, but acts also as an artificial stem cell niche containing haematopoietic and multipotent cells, similarly to bone marrow and perivascular niches. Our *in vitro* model provides the first evidence that multipotent cells could be mobilized to peripheral blood under physiological conditions and not only under stress conditions (*i.e*. inflammation, tissue damage, stimulation by drugs or growth factors), as commonly reported. Likely the haematopoietic stem cell niche, CPL‐MB results as a complex milieu that regulates, by structural and bioactive factors, the survival, expansion, differentiation and transendothelial migration of immature cells. Although a growing body of evidence suggests the existence of multipotent cells in peripheral blood, to date, the use of blood as an alternative source of autologous stem cells in regenerative medicine is limited by several important questions concerning the predictability of successful isolation and *ex vivo* expansion by a standardized protocol. Produced according to Italian standards of quality assurance and Caloprisco's method, CPL‐MB could represent a valid strategy to bypass the intrinsic heterogeneity of blood samples and to normalize the cell content of blood derivatives for obtaining autologous cells with a defined stemness signature.

## Conflict of interest

The authors indicated no potential conflict of interests.

## Author contributions

D.L.R. and P.P.P.: involved in the study conception and design; D.L.R.: involved in the data analysis and interpretation, manuscript writing and final approval of manuscript; B.T. and S.S.: involved in the collection and assembly of data and contributed to manuscript writing; B.A., P.I. and C.S.: contributed to the preparation and quality control of CLP‐MB; A.A.: contributed to scientific and financial support; C.C.: contributed to collection and assembly of data; and C.M.T. and P.P.P.: contributed to data interpretation and final approval of manuscript.
